# Comparing Molecular Variation to Morphological Species Designations in the Deep-Sea Coral *Narella* Reveals New Insights into Seamount Coral Ranges

**DOI:** 10.1371/journal.pone.0045555

**Published:** 2012-09-27

**Authors:** Amy R. Baco, Stephen D. Cairns

**Affiliations:** 1 Associated Scientists at Woods Hole, Woods Hole, Massachusetts, United States of America; 2 Department of Invertebrate Zoology, Smithsonian Institution, NMNH W-326, MRC-163, Washington, D.C., United States of America; University of Canterbury, New Zealand

## Abstract

Recent studies have countered the paradigm of seamount isolation, confounding conservation efforts at a critical time. Efforts to study deep-sea corals, one of the dominant taxa on seamounts, to understand seamount connectivity, are hampered by a lack of taxonomic keys. A prerequisite for connectivity is species overlap. Attempts to better understand species overlap using DNA barcoding methods suggest coral species are widely distributed on seamounts and nearby features. However, no baseline has been established for variation in these genetic markers relative to morphological species designations for deep-sea octocoral families. Here we assess levels of genetic variation in potential octocoral mitochondrial barcode markers relative to thoroughly examined morphological species in the genus *Narella*. The combination of six markers used here, approximately 3350 bp of the mitochondrial genome, resolved 83% of the morphological species. Our results show that two of the markers, ND2 and NCR1, are not sufficient to resolve *genera* within Primnoidae, let alone species. Re-evaluation of previous studies of seamount octocorals based on these results suggest that those studies were looking at distributions at a level higher than species, possibly even genus or subfamily. Results for *Narella* show that using more markers provides haplotypes with relatively narrow depth ranges on the seamounts studied. Given the lack of 100% resolution of species with such a large portion of the mitochondrial genome, we argue that previous genetic studies have not resolved the degree of species overlap on seamounts and that we may not have the power to even test the hypothesis of seamount isolation using mitochondrial markers, let alone refute it. Thus a precautionary approach is advocated in seamount conservation and management, and the potential for depth structuring should be considered.

## Introduction

Distance, hydrography, and life history strategies contribute to the isolation of seamount fauna. Because of this isolation, seamounts are often cited as potential locations for high levels of speciation [1]–[6] and a large percentage of the fauna that have been studied on seamounts were found to be endemic [4]–[6]. More recently, studies have found high levels of species overlap and low levels of genetic differentiation among seamount locations [7]–[12] calling into question the base theory of seamount isolation.

These confusing results regarding isolation of seamounts may hamper conservation efforts at a critical time. The benthic fauna of seamounts are currently under threat from trawl and long-line fisheries [4], [6] and from proposed cobalt- manganese crust mining [13], [14]. Practices that remove the benthic fauna in the path of the trawl [15], [16], or in the case of mining, will remove the entire benthic substrate. Given the active and potential threats to seamount fauna, there is a need for a better understanding of the connectivity of seamount fauna for conservation and management purposes.

Deep-sea corals are an ideal group to focus on for improved understanding of seamount ecology because much of the benthic habitat of seamounts is hard substrate and a large percentage of the fauna are suspension feeders, with gorgonian octocorals and antipatharians often dominating the communities numerically [4], [17] and in biomass [18]. Corals are also an important component of seamount biodiversity. A review of records of all faunal groups in the Seamounts Online database indicated that corals are also one of the most diverse group of invertebrates found on seamounts [19]. Thus the abundance, numerical dominance, high biomass, and diversity of deep-sea corals make them ideal model organisms to study seamount ecology.

However, recent reviews of seamount fauna, deep-sea corals, and deep-sea corals on seamounts have all cited a “global deficiency of scientific expertise in [morphological] taxonomy” [20] as a significant impediment to our understanding of deep-sea coral diversity, coral biogeography, and seamount ecology [20]–[24]. For example, identification based on morphology for many deep-sea octocoral families in the North Pacific, particularly the bamboo corals in the family Isididae, and also the Plexauridae, is hindered by lack of sufficient keys, or molecular characters which conflict with morphological designations of genera [25], there are also a number of undescribed species in the Coralliidae [26] and Paragorgiidae [27] (Sanchez unpubl data). As these are some of the dominant deep-sea families, currently less than 50% of specimens can be identified to species level based on morphology. This provides a significant challenge for determining whether species present on a given seamount are the same as species at another location, a prerequisite for assessing levels of connectivity between sites. Molecular genetic methods can be used to more rapidly assess species identifications as well as diversity in archived specimen collections [28]–[30]. These genetic methods are only recently being applied to deep-sea corals [7], [10], [31], [32].

One of the first attempts at “barcoding” deep-sea octocorals using genetic markers was on bamboo corals from trawls off New Zealand [7]. Because these fragmented specimens could not be identified morphologically, two mitochondrial genetic markers were used to assess the distribution of purported species based on the distribution of haplotypes for the two markers. The results of this study indicated that both “species” and haplotypes were widespread; however, there was no baseline for the level of variation in these markers relative to what constitutes a morphological species, i.e., haplotypes for these markers could correspond to any taxonomic level, e.g. anywhere from subspecies to genera or higher. Although this major caveat was clearly stated in the paper, the Smith *et al.* [7] results have been widely cited as indicative of seamount species being widespread and not as isolated as previously thought.

One of the only other efforts to use genetic barcodes to examine seamount octocoral species distributions was the study by Thoma *et al.* [10] that focused on a third mitochondrial gene, MutS. In their paper they stated that they were making the assumption that each unique MutS haplotype corresponds to a species, however they provide the same caveat, that it is not clear that MutS haplotypes correspond to morphological species [10]. Despite this caveat, the study concluded that, based on widespread MutS haplotypes, seamount “species” of octocorals were also widespread.

Thus, although the existing octocoral barcoding studies support widespread distribution of octocoral species and haplotypes, providing the first stage of support for seamount connectivity, it is difficult to draw solid conclusions from them about the true distribution of octocoral species on seamounts, given the lack of a well-defined baseline of variation in the markers that were used as barcoding proxies. Most recently, a paper by McFadden *et al.* [24] begins to address this issue, by focusing on a combination of three markers as a potential barcode for octocorals, making it one of the first papers to tie levels of genetic variation within potential barcoding markers for octocorals, with thoroughly examined morphological species within the same genus. However, the morphological work in their paper focuses on shallow-water Family Alcyoniidae. The species represented in the deep sea come from a different group of families than what are found in shallow water. Although there are a number of deep-sea samples in the McFadden *et al.* [24] study, they are not included in the in-depth morphological assessment.

Thus, the goal of our study is to use a group of deep-sea octocorals, in the primnoid genus *Narella*, collected intact from a number of seamount sites in the Pacific, to compare levels of genetic variation in a suite of potential octocoral barcoding markers, to morphological species designations. *Narella* is a species-rich genus that was chosen as a target taxon because it has adequate keys and taxonomic expertise for resolution to the morphological species level. The results of this marker-testing exercise are then extended to examine the distribution of species and haplotypes on seamounts in the North Pacific. We also reconsider the results of previous seamount octocoral studies in light of levels of genetic variation relative to species designation as suggested by our study.

## Methods

### Collections

**Figure 1:**
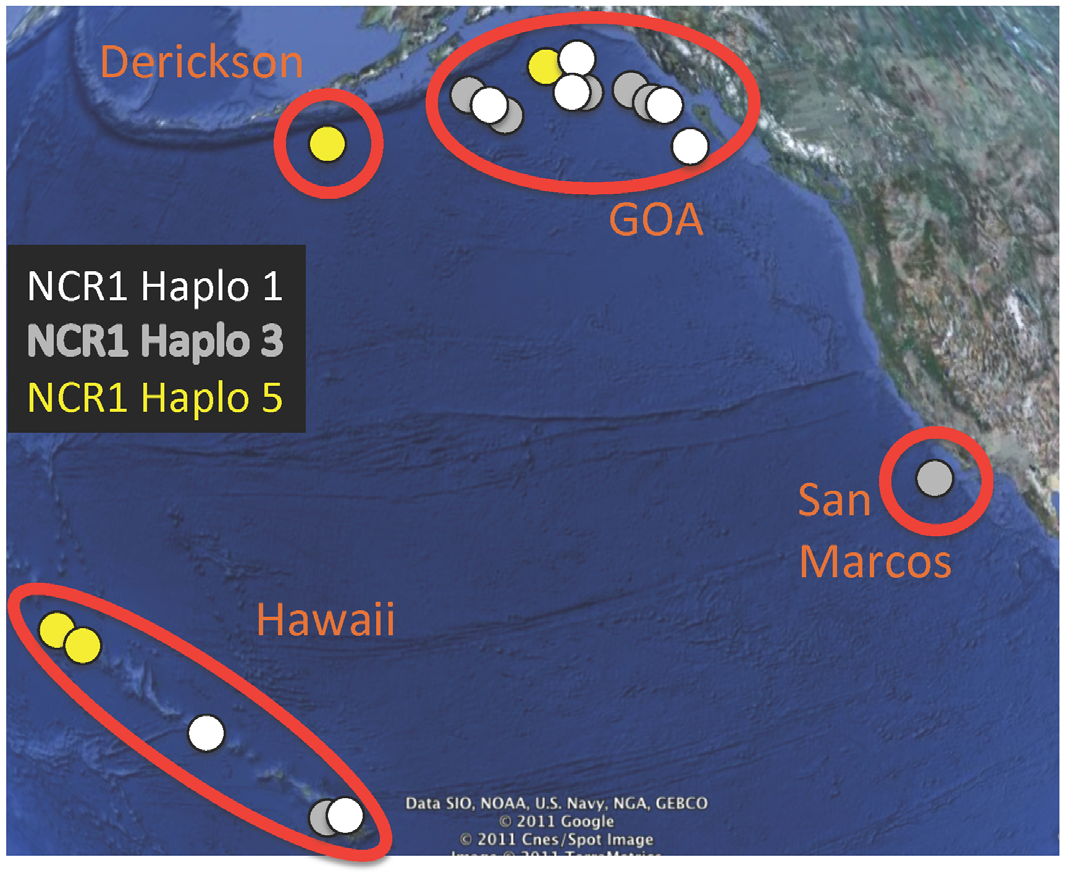
Map indicating regions of seamount collections. Overlaid is the distribution of haplotypes for the NCR1 gene. For clarity, only the three most common haplotypes are shown. Haplotype 1 occurred in *Narella* in Hawaii, but only in *Parastenella* and *Primnoa* in the GOA. Satellite imagery: GoogleEarth. Date accessed: 05 Jan 2011. Co-ordinates: approx. 18 to 61°N, 168°E to 114°W.

A total of 41 specimens of *Narella* plus a suite of additional primnoid specimens from the same locations (Table 1) were included in the analyses for intergeneric comparisons. All specimens were collected from 1998–2004 from four regions of seamounts in the North Pacific (Figure 1, Table 1). Samples in the Hawaiian Archipelago were collected with the *Pisces 4* and *Pisces 5* submersibles, from the Gulf of Alaska (GOA) Seamounts using the *Alvin* submersible, from Derickson Seamount using the ROV *Jason II* and from San Marcos Seamount using the ROV *Tiburon*. All necessary permits were obtained for the described field studies. Corals collected in the Northwestern Hawaiian Islands were collected when it was the NWHI Coral Reef Ecosystem Reserve under permit # NWHICRER-2003-003 and -004. Collections were made prior to establishment of the Papahanaumokuakea Monument. There is no permit required for collection of specimens in Alaskan waters. CITES does not apply to octocorals, and all collections were made in US waters and stayed in the US.

**Table 1:** Collection information for the specimens used in this study.

			ID						Haplotype^8^										
USNM #	Published ID		In		Dive #	Site		Depth	N1	N6		N2		CI		MS		Comb	
–	*Narella alaskensis* Cairns & Baco, 2007		1		AD-3797	Murray		2254?	3	3		3		3		3		**1**	
1075468	*Narella alaskensis* Cairns & Baco, 2007		3		AD-4027	Denson		2377	3	3		3		3		3		**1**	
–	*Narella alaskensis* Cairns & Baco, 2007		1		AD-3797	Murray		2511	3	3		3		3		3		**1**	
1075471	*Narella alaskensis* Cairns & Baco, 2007		3		AD-4033	Welker		2634	3	3		3		3		3		**1**	
1080453	*Narella alaskensis* Cairns & Baco, 2007		3		AD-3797	Murray		2680?	3	3		3		3		3		**1**	
1075469	*Narella alaskensis* Cairns & Baco, 2007		3		AD-4029	Dickins		2736	3	3		3		3*		3		**1**	
–	*Narella alaskensis* Cairns & Baco, 2007		1		AD-3797	Murray		2264–2680′	3	3		3		3		3		**1**	
Acc2042645	*Narella alaskensis* Cairns & Baco, 2007		1		T669-A6	San Marcos		2050	3	3		3		3		3		1	
1080454	*Narella alaskensis* Cairns & Baco, 2007		3		AD-3803	Chirikoff		3075	3	3		20*		3		3		**4**	
1072109	*Narella hawaiiensis* Cairns & Bayer, 2008		4		P5-526	Pioneer		1743.7	9*	26		3		3		9*		**2**	
1071215	*Narella hawaiiensis* Cairns & Bayer, 2008		4		P5-594	Keahole		*400–1492*′	3*	26		15*		15		15*		**3**	
1072111 or 1072112	*Narella dichotoma Cairns & Bayer, 2008*		4		P5-527	Pioneer		*1209.0*	12	12		12		3		12		**5**	
1072111 or 1072112	*Narella dichotoma Cairns & Bayer, 2008*		4		P5-527	Pioneer		*1209.0*	12*	12*		12		3		12		**5**	
1071422	*Narella dichotoma Cairns & Bayer, 2008*		4		P5-593	Keahole		*743.21*	12*	19*		12		3*		19*		**6**	
1071418	*Narella alata Cairns & Bayer, 2008*		4		P5-595	Keahole		*679.44*	1	1		1		1		1		**17**	
1071421	*Narella alata Cairns & Bayer, 2008*		4		P5-593	Keahole		*749.56*	1	1		1		1		1		**17**	
1071420	*Narella alata Cairns & Bayer, 2008*		4		P5-593	Keahole		*749.56*	1	1		1		1*		1		**17**	
1071420	*Narella alata Cairns & Bayer, 2008*		4		P5-593	Keahole		*749.56*	1	1		1		1		1		**17**	
1071419	*Narella alata Cairns & Bayer, 2008*		4		P5-593	Keahole		*749.56*	1	1		1		1		1		**17**	
1072131	*Narella sp. 1 (morph closest to dichotoma)*		2		P5-544	E of Necker		*1812.6*	1*	1		8*		8		8*		**18**	
1080450	*Narella abyssalis Cairns & Baco, 2007*		3		JD-093	Derickson		*4594*	2	2		2		27		2		**8**	
1080447	*Narella bayeri Cairns & Baco, 2007*		3		JD-093	Derickson		*3292*	2*	2		2		2*		2		**9**	
1080448	*Narella bayeri Cairns & Baco, 2007*		3		JD-091	Aleutian Slope		*3277*	21	2		2		2*		2		**10**	
1080446	*Narella bayeri Cairns & Baco, 2007*		3		JD-093	Derickson		*4091*	21*	2		2		2*		2		**10**	
1080449	*Narella cristata Cairns & Baco, 2007*		3		JD-093	Derickson		*3385*	21	2		2		21		2*		**11**	
1072118	*Narella macrocalyx Cairns & Bayer, 2008*		4		P5-534	SE of Laysan		*1206.0*	11	5		5		11		11		**12**	
1072133	*Narella macrocalyx Cairns & Bayer, 2008*		4		P5-544	E of Necker		*1443.4*	11	5		5		11		11		**12**	
1072122	*Narella macrocalyx Cairns & Bayer, 2008*		4		P5-542	E of Necker		*1697.4*	11	5		5		11		11		**12**	
1072105	*Narella macrocalyx Cairns & Bayer, 2008*		4		P5-525	Pioneer		*1706.0*	11	5		5		11		11		**12**	
1072116	*Narella sp. 2*		2		P5-532	SE of Laysan		*1807.0*	5*	5*		4*		5		4		**13**	
1072108	*Narella sp. cf. macrocalyx*		2		P5-526	Pioneer		*1743.7*	5	5*		5		5		4		**15**	
1072103	*Narella sp. cf. macrocalyx*		2		P5-525	Pioneer		*1802.0*	5*	5*		5*		5*		4*		**15**	
1072117	*Narella (sp. cf.) macrocalyx Cairns & Bayer, 2008*		3, 7		P5-532	SE of Laysan		*1807.0*	5	5		5		5		4		**15**	
1080452	*Narella arbuscula Cairns & Baco, 2007*		3		JD-093	Derickson		*3465*	5	5		5		5*		4*		**15**	
1080451	*Narella arbuscula Cairns & Baco, 2007*		3		JD-093	Derickson		*2775*	5	5		5		4		4		**14**	
1075465	*Narella arbuscula Cairns & Baco, 2007*		3		AD-4041	Giacomini		*2818.6*	5	5		5		5		5		**16**	
–	*Narella sp.*		1		AD-4041	Giacomini		*2818.6*	5	5		5		5		5		**16**	
1075466	*Narella arbuscula Cairns & Baco, 2007*		3		AD-4041	Giacomini		*2818.8*	5	5		5		5		5		**16**	
1075467	*Narella arbuscula Cairns & Baco, 2007*		3		AD-4041	Giacomini		*2810–2818′*	5	5		5		5		5		**16**	
1154063	*Callogorgia gilberti*		2		P5-359	Makapuu		*411.3*	10	10		10		10		10		**7**	
1075379	*Parastenella ramosa*		2		AD-4039	Pratt		*918*	1*	17*		17		17		17*		**19**	
1082620 or 1082624	*Parastenella gymnogaster*		6		AD-3804	Marchand		*2417*	1	17		2		17*		23		**20**	
–	*Parastenella sp.*		1		AD-4035	Welker		*1084*	1*	22		2		17		22		**21**	
1082639	*Parastenella ramosa*		6		AD 3806	Warwick		*808–872′*	1	22		2		17*		22		**21**	
1075478	*Primnoa pacifica willeyi Hickson, 1915*		6		AD-4028	Dickins		*755*	1*	7		7		7		7*		**22**	
1082615	*Calyptrophora laevispinosa*		6		AD-3802	Patton		*1834/1778′*	6	6		6		6*		6		**23**	
1082617	*Calyptrophora laevispinosa*		6		AD-3802	Patton		*1993*	6	6		6		6*		6		**23**	
1075472	*Calyptrophora laevispinosa*		6		AD-4033	Welker		*2757*	6	6		6		25		6		**24**	
1082616	*Calyptrophora laevispinosa*		6		AD-3802	Patton		*1778–3075′*	6	6		6		25*		6		**24**	
1071947	*Calyptrophora wyvillei Wright 1885*		5		P5-524	Pioneer		*1200*	18	18		6		18		18		**25**	
1071423	*Calyptrophora wyvillei Wright 1885*		5		P5-595	Keahole		*935*	18*	18		6		18		18		**25**	
1072130	*Calyptrophora wyvillei Wright 1885*		5		P5-543	E of Necker		*1278*	13	13		13		13		13		**26**	
1072135	*Paracalyptrophora hawaiiensis Cairns 2009*		5		P5-545	Twin Banks		*407*	14	14		14		14*		14		**27**	
1071245	*Paracalyptrophora hawaiiensis Cairns 2009*		5		P5-587	Cross		*388.71*	14	14		14*		14*		14		**27**	
Specimen Identified In																			
1		Material not examined morphologically, generally small recruit used up for DNA extraction																	
2		Cairns unpublished identification																	
3		[33]																	
4		[34]																	
5		[54]																	
6		[36]																	
7		New identification based on reexamination for this study																	
8		N1 = NCR1, N6 = ND6, N2 = ND2, CI = COI+, MS = MutS																	

USNM# is the catalog number for the specimen at the Smithsonian Institution. Dive numbers are abbreviated by vehicle – AD- Alvin Dive, P5– Pisces 5, T – Tiburon, JD – Jason II Dive. Haplotypes for each marker are number coded, with each number indicating a unique sequence for a given marker. Variable positions among haplotypes of Narella for each marker are given in Supplemental Table 2.

‘– specimen depth not recorded, values given are for depth range for dive, or specimens collected before and after that individual.

* indicates a specimens which was sequenced more than once, with identical sequences encountered every time.

Across vehicle types, samples were collected from the seafloor using manipulators and placed into bioboxes for return to the surface. Samples were immediately preserved at −80°C and/or in ethanol for genetic analyses and in ethanol for morphological analyses. The exact specimens that were sequenced were examined for morphology by SC prior to viewing the molecular results, with morphological species designations and descriptions in Cairns & Baco [33], Cairns & Bayer [34], and Cairns [35], [36].

### Choice of Mitochondrial Markers

France & Hoover [37] examined a number of deep-sea octocorals and established that MutS (also referred to in much of the octocoral literature as Msh1, but reestablished as MutS by Bilewitch and Degnan [38]) has the highest rate of substitution of any mitochondrial gene studied in octocorals to that point. They indicate this gene may be useful for molecular phylogenetic analyses of octocorals ranging from “intrageneric to interordinal levels”, however most comparisons in the paper were not among species within the same genus. This gene is the most widely used marker for octocoral genetics, including for phylogenetics [39]; as an aid in morphological species descriptions [33], [40], [41]; and as a barcoding proxy [10], [24].

The NADH subunits ND6 and/or ND2 have often been used in combination with MutS in octocorals for phylogenetics and species descriptions [33], [39]–[41]. McFadden *et al.* [42] also used several NADH subunits (without MutS) and found that they were sufficient to resolve phylogenetic relationships among families and some genera in the shallow water families Alcyoniidae and Xeniidae. All of these studies conclude that the best approach for intraspecific through intrageneric studies of octocorals would be a combination of MutS and NADH genes.

The markers of the Smith *et al.* [7] study for bamboo octocorals in the family Isididae include a portion of the 16S rRNA gene [43], here referred to as NCR1 and a non-coding region between the mitochondrial COI and COII genes [7], further referred to as NCR2. NCR1 was found to be more variable than NCR2, however, the range of variation in the two markers of the Smith *et al.* [7] study was not compared to the range of variation in MutS or to the NADH markers for the same specimens.

Recently a study by McFadden *et al.* [24] evaluated the “Folmer region” of mitochondrial COI and an intergenic region adjacent to COI that corresponds to NCR2, (amplified with a single primer pair and referred to together in this paper as COI+) along with MutS, as barcoding proxies for octocorals. They conclude that the combination of MutS, COI and the intergenic region provide the best resolution of any markers for octocorals, but NADH markers and the NCR1 of the Smith *et al.* [7] study were not included in their analyses. They also found that even the combination of three markers was not sufficient to resolve all morphological species.

Of these studies, most focus on shallow-water octocorals, and the only of these studies to look at more than two species within a given genus also focus on shallow-water corals, which represent a different suite of families than is found in the deep sea. None of these studies have compared the full suite of available markers for octocorals in the same specimens. Thus, here we focus on a genus in one of the more common deep-sea families, the Primnoidae, and use all of the aforementioned markers in the same specimens, in the genus *Narella*, and combine these results with morphological species identifications, to ground-truth levels of variation that correspond to morphological species. We also attempted to evaluate ITS2 and SRP 54, two nuclear introns that have been used in octocorals [44], [45]. ITS2 was multicopy for all amplifications, including annealing temperatures up to 58°C, and therefore is not further discussed in the text. Despite many attempts using a variety of PCR conditions, we could not get SRP54 to amplify in these or several other families of octocorals we tried, so it also not discussed in the text.

### Extraction, PCR and Sequencing

A small portion (1–5 polyps depending on size) of tissue from each specimen was used for genomic DNA extractions using a Qiagen DnEasy animal kit (Qiagen). An Eppendorf MasterCycler epgradient (Eppendorf) thermocycler was used to carry out PCR amplifications (primers and conditions in Table 2) of 50 µl final volume with approximately 50 ng of DNA, 1X PCR Buffer (Promega), 2.5 mM MgCl_2_, 1 mM dNTPs, 1 µM of each primer and 1.5 U Taq polymerase (Promega). Part-way through the project we switched to GoTaq (Promega) using the supplied green buffer. This buffer contains 7.5 mM MgCl_2_, which in a 50 µl reaction (1∶5 dilution) gives a concentration of 1.5 mM. There was no noticeable difference in PCR results or need for PCR condition changes with the new Taq and buffer. PCR products were purified and sequenced at the University of Washington High-Throughput Genomics Unit. Sequences were cleaned, aligned, and haplotypes assigned using Sequencher 4.8 (Gene Codes Corp.). Genetic distances, as uncorrected ‘p’ distances, between haplotypes were calculated using PAUP v.4.0b10 [46]. Only specimens for which all markers amplified are included in the results and discussion. GenBank numbers for each haplotype for each marker are includes in Table S1.

**Table 2:** Primers used for this study.

Target Marker	Primer Name	Primer Sequence	Denat.	Annealing	Cycles	Reference
			Temp	Temp		
			Time	Time		
MutS	ND42599F	GCCATTATGGTTAACTATTAC	94°C	57°C	**35**–40	[37]
	MUT-3458R	TSGAGCAAAAGCCACTCC	**60**–90s	45–**60**s		[37]
ND2	16S-647F	ACACAGCTCGGTTTCTATCTACAA	94°C	52–**53**°C	**35**–40	[42]
	ND2-1418R	ACATCGGGAGCCCACATA	90s	90s		[42]
ND6	ND6-1487F	TTTGGTTAGTTATTGCCTTT	94°C	40–48°C; **47**	40	[42]
	ND3-2126R	CACATTCATAGACCGACACTT	**90**–115s	90s		[42]
COI+	HCO2198	TAAACTTCAGGGTGACCAAAAAATCA	94°C	46–**48**°C	40	[78]
	COII-8068F	CCATAACAGGACTAGCAGCATC	60s	**60**–90		[42]
NCR1	OCTO1_L	AGACCCTATCGAGCTTTACTGG	94°C	55°C	35	[43]
	OCTO2_H	CGATAAGAACTCTCCGACAATA	30s	40s		[43]

COI+ includes the “Folmer reigon” of COI along with the intergenic spacer between COI and COII, which is NCR2 of Smith *et al.* [7] and is coded as *igr1* in McFadden *et al.* [24]. All PCRs began with a 2-min hot start at 94–96°C followed by the denaturing and annealing protocols below. Each cycle was completed with a 1 min at 72°C step (45 s for OctoH/L primers) for the number of cycles listed in the Table and after all cycles, a single round of 72°C for 5 mins followed by a 4°C hold. Where conditions are variable, conditions used most often are in bold, with adjustments to time and temps to get stragglers also shown.

In most taxa, mitochondrial DNA is inherited as a single locus. As all included makers were mitochondrial, a combined sequence was made for each individual with all markers for that individual. These are referred to throughout the text as “combination haplotypes” and were constructed and aligned in Sequencher 4.8 (Gene Codes Corp.) and analyzed in the same manner as the individual markers.

### Error Check

Because of the possibility of error during PCR and the low sequence variation between haplotypes, in many cases only 1–2 bp, a random selection of samples were re-PCR’d on a second PCR machine in a different lab. In all cases, an identical sequence was obtained.

For all cases in which more than 1 haplotype was found for different individuals of a morphological species, the specimens were reexamined to determine if any characters might distinguish the second haplotype as a new species.

### Phylogenetic Analyses

Sequencher 4.8 (Gene Codes Corp.) was also used to identify nucleotide characters and haplotypes diagnostic for a given species. Phylogenetic reconstructions, based on the combination haplotype sequences, used a TPM1uf+I+G model, as selected with the AIC criterion in the program jModelTest 0.1.1 [47], were run in PAUP v.4.0b10 [46]. PAUP v.4.0b10 [46] was also used to construct a maximum likelihood tree using the jModelTest results and to obtain maximum likelihood bootstrap values. For base likelihood topology, a heuristic search with random addition sequence was used for 100 replicates. For bootstrap values, one replicate for each of 1000 bootstraps was used. Mr. Bayes 3.1.2 [48], [49] was used to construct a Bayesian tree using the same jModelTest parameters. The following additional parameters were used in the Bayesian analyses; 2 runs, 4 chains, 4 million generations, sample frequency of 100 and burn-in of 10,001. Clades were considered supported if they had likelihood bootstrap values ≥70% [50] and Bayesian posterior probabilities ≥95% [51], [52]. We tested for monophyly of *Narella* using the Shimodaira-Hasegawa test [53] in PAUP v.4.0b10 [46]. For this test maximum likelihood trees were generated from a heuristic search with *Narella* monophyletic as a topological constraint, and compared to the tree that resulted from the unconstrained heuristic search outlined above.

## Results

Based on morphological taxonomy, the 41 specimens of *Narella* separated into 12 species or morphotypes [33]–[36], [54] (and Cairns unpublished data), listed in Table 1 with their published identification and USNM catalog number. The presence of indels within the sequences was variable by marker, but all were easily alignable by eye except a portion of a gap within the NCR1 marker. All alignable gaps were kept in for the analyses. Most gaps were for comparisons of *Narella* to outgroup taxa or among outgroup taxa.

The NCR1 marker was 306 nt long once aligned, with an 31 nt indel region present with one set of gaps present in *Paracalyptrophora*, and *Calyptrophora*, and a different set of gaps in the same region in *N. bayeri*, *N. abyssalis* and *N. cristata*, from Derickson seamount. Out of this indel region a 26 nt portion was not alignable and so was not included in the analyses for this marker, making the final analyzed length 280 nt.

The ND6 marker had no indel regions and was 606 nt in length across taxa. The ND2 alignment was 775 bp long and includes portions of both the 16S gene and the ND2 gene. Within the 16S portion of this marker a 28 nt indel region produced gaps in the genera *Callogorgia, Paracalyptrophora*, and *Calyptrophora*, but not for the other genera. There was a 1 base insertion in all haplotypes of *Paracalytrophora* and *Calyptrophora* just before this gap, but the intervening sequence aligned perfectly with no base changes. A second 12 nt indel in the 16S portion produced gaps in both haplotypes for *N. alaskensis*, and all but one haplotype of *Parastenella* (Haplotype 17).

The COI+ alignment was 877 nt long once uneven ends were trimmed. The only indels fell into the intergenic spacer region and consisted of a 20 nt gap in *Callogorgia* and a 13 nt gap in *N. abyssalis.* The MutS marker was 854 nt long with base insertions in the non-coding region of the marker at positions 15 and 32 from the end of the ND42599F primer in both individuals of *Paracalyptrophora*. There was also an ambiguous base at position 35 in about half the individuals; this position was not included in the analyses. The only indel in the coding region of the marker was a gap in *Callogorgia* at positions 802–810 and did not shift the amino acid reading frame for this sequence.

The final combination of all markers with uneven ends, unalignable gap in NCR1, and 1 ambiguous position of MutS removed, was 3348 nt.

### Evaluation of Markers

McFadden *et al.* [24] provide an in-depth comparison of the various means of evaluating markers as barcoding proxies for octocoral species rooted in levels of pairwise intraspecific distances. They show that, because of the low levels of variation in octocoral markers, in fact a character-based approach, comparable to Smith *et al.* [7], Thoma *et al.* [10] and formalized in DeSalle et al [55] and Rach *et al.* [29], was a better way to resolve species for octocorals. This method is also supported as preferable over distance-based approaches in a recent review of barcoding across taxa [56]. Thus our explanation of results focuses on a character-based approach rather than distance-based using haplotypes to identify unique characters. We define haplotypes within each marker as sequences having at least 1 nt difference or a gap difference from all other haplotypes for that marker, with variable sites provided for *Narella* in Table S2.

Of the markers of this study, in terms of the number of haplotypes, MutS was the most variable of the markers, as had been found previously [24], [37] and the COI+ marker suggested as an extended barcode with MutS by McFadden *et al.* [24] was the second most variable (Table 3). The two NADH regions were the next most variable, and finally, the NCR1 marker, which was the more variable of the two markers in the Smith *et al.* [7] study, was the least variable of all the markers studied here (Table 3). (NCR2, the other marker used by Smith et al [7], was not evaluated without the COI gene it was amplified with, since it was already shown by itself to be less variable than NCR1 in the Smith *et al.* [7] study). In fact, levels of variation in NCR1 were not high enough to distinguish among the subset of primnoid genera included in this study, with NCR1 Haplotype 1 shared between *N. alata, N. sp.1, Primnoa* and *Parastenella* (Table 1). The ND2 marker was also not variable enough to resolve genera, with “resolve” here defined as haplotypes within a given taxon being exclusive to that taxon. In the case of ND2, Haplotype 2 for that marker was shared between *Narella* and *Parastenella* (Table 1). The remaining markers had 1–7 nucleotide changes between genera.

**Table 3:** Summary statistics for each marker.

	NCR1	ND6	ND2	COI+	MutS	Combo	COI+ andMutS	COI+, MutSand ND2
**Analyzed sequence length**	280	608	771	836	853	3348	1689	2460
**Number of haplotypes**	13	15	15	18	20	27	24	26
**Number of ** ***Narella*** ** haplotypes**	8	7	9	10	11	17	14	16
**Number of the 12 ** ***Narella*** ** morphotypes fully resolved**	2	3	4	6	6	10	9	10
**% resolved**	17	25	33	50	50	83	75	83
**Intraspecific Variation** **Range**	0–0.36%	0–0.50%	0–0.13%	0–0.24%	0–0.47%	0–0.21%	0–0.30%	0–0.25%
**Corresponding #** **of bp changes**	0–1	0–3	0–1	0–2	0–4	0–7	0–5	0–6
**Interspecific Variation** **Range**	0–1.79%	0–0.83%	0–0.79%	0–1.20%	0–1.41%	0–0.81%	0–1.07%	0–0.94%
**Corresponding #** **of bp changes**	0–5	0–5	0–6	0–10	0–12	0–27	0–18	0–23
**Intergeneric Range**	**0–**2.14%	0.17–1.98%	**0–**1.75%	0.8–2.82%	0.35–3.52%	0.33–2.37%	0.48–3.08%	0.37–2.63%
**Corresponding #** **of bp changes**	**0**–6	1–12	**0**–14	7–24	3–30	11–79	8–52	9–65

Sequence length indicates final length of the alignment after the ends were trimmed and unalignable gaps were removed as outlined in the text. “Resolved” refers to the species for which all observed haplotypes for a species for the given marker were unique to that species. Distances are given as uncorrected “p” distances, with the number of base changes the distance values correspond to given in the subsequent line.

At the intraspecific level, each of the markers had some species that had more than one haplotype, with anywhere from 1–4 differences in those haplotypes depending on the marker. However, in many cases these were not unique to the species. For example, *N. hawaiiensis* had two haplotypes for NCR1, but one of the haplotypes was shared with *N. alaskensis*. ND6 on the other hand had two haplotypes that were unique to *N. dichotoma*. MutS had two haplotypes that were unique to *N. hawaiiensis*, and two unique to *N. dichotoma*. The combination haplotypes showed at least three species that have more than one unique haplotype for a given species. In no case in any marker was the genetic distance among intraspecific haplotypes greater then the 0.5% criteria suggested as indicative of cryptic species cutoff by McFadden *et al.* [24].

### Haplotype Distribution with Depth and Distance

In the interest of examining the distribution of these corals on seamounts, we examined the depth and geographic distribution of each of the haplotypes for each of the markers, as well as for the combination haplotypes.

The NCR1 marker was the least variable of the markers. It had eight haplotypes for *Narella*. Of these, two haplotypes were particularly widespread (Figure 1), covering the geographic range of sampling. NCR1 Haplotype 5 was the most widespread, occurring in the Northwestern Hawaiian Islands (NWHI), Derickson Seamount in the Aleutians, and in the GOA seamounts. NCR1 Haplotype 3 occurred in the GOA, one site in Hawaii, and off of southern California on San Marcos Seamount. A third haplotype, Haplotype 1 was also found in two species of *Narella* in Hawaii, along with *Parastenella* and *Primnoa* from the GOA (Figure 1). The remaining haplotypes for NCR1 were restricted to a given seamount chain.

**Figure 2:**
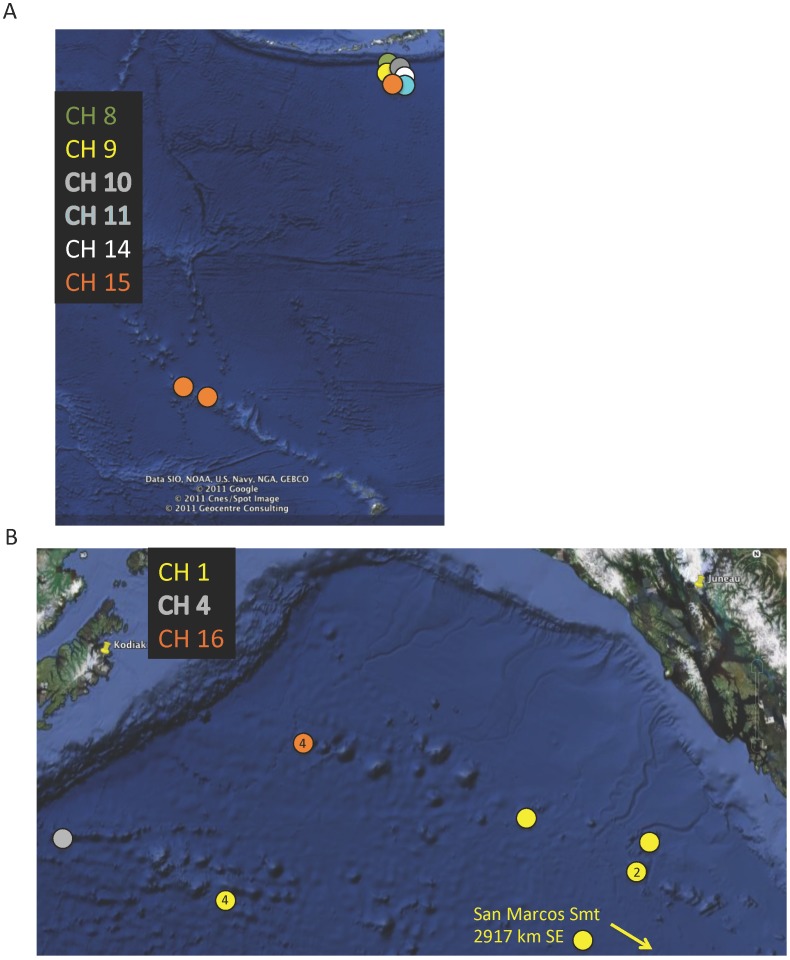
Geographic distribution of combination haplotypes found in Alaskan waters. A. Distribution for combination haplotypes that occur on Derickson Seamount. No combination haplotypes on Derickson were shared with the GOA Seamounts, overlap with Hawaii is shown. Satellite imagery: GoogleEarth. Date accessed: 05 Jan 2011. Co-ordinates: approx. 18–58°N, 168°E to 150°W. B. Geographic distribution of combination haplotypes found in the Gulf of Alaska. Numbers within a circle indicate number of individuals with that haplotype when greater than 1 for a feature. Satellite imagery: GoogleEarth. Date accessed: 05 Jan 2011. Co-ordinates: approx. 52° to 59°30*′*N, 153°50*′* to 143°20*′*W.

**Figure 3:**
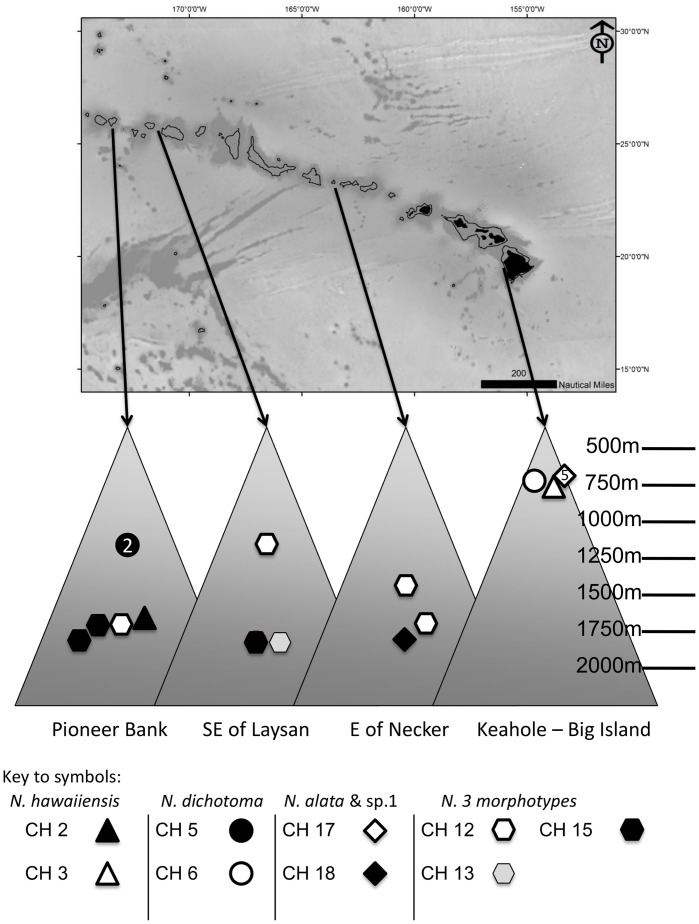
Geographic distribution of combination haplotypes found in the Hawaiian Archipelago. The upper panel provides a geographic context for the features of interest. Land is black and the 1000 m isobath is provided. The lower panel provides a depth scale to demonstrate the difference in depth range between haplotypes of a given morphotype(s). Base map prepared in ESRI ArcMap 10.0 using bathymetric data from GEBCO http://www.gebco.net/(30 arc second version) and terrestrial data from http://www.naturalearthdata.com/. Accessed 01 July 2011.

The ranges of the combination haplotypes were much more restricted (Figure 2,3). On a regional basis, Derickson Seamount had 6 haplotypes. Five were restricted to this seamount and one was shared with two sites in the NWHI (>3100 km straight line distance) (Figure 2A). Although Derickson is geographically closer to the GOA seamounts than to the NWHI, no combination haplotypes were shared between Derickson and the GOA seamounts.

Within the GOA seamount chain (Figure 2B), three combination haplotypes were present representing two species, *N. arbuscula* and *N. alaskensis*. The *N. arbuscula* haplotype was restricted to Giacomini seamount, and all four *Narella* specimens from this seamount had the same haplotype. Of the *N. alaskensis* haplotypes, Haplotype 4 was restricted to Chirikoff Seamount, Haplotype 1 occurred on several GOA seamounts as well as on San Marcos Seamount, off southern California. Chirikoff Seamount was the farthest west of the seamounts *N. alaskensis* was collected from. The distance between Chirikoff and the nearest other site for *N. alaskensis* (311 km) is less than the distance between the nearest GOA Seamount and San Marcos (2917 km) though. However, the specimen taken from Chirikoff was collected from 3075 m, about 300 m deeper than it was collected from any other site.

While a smaller number of specimens came from Hawaii compared to the Alaskan sites (17 from Hawaii vs. 20 from Derickson and GOA together), a greater number of combination haplotypes were present. The higher haplotype diversity occurs largely because most morphological species have greater than one haplotype. Interestingly, in the case where two haplotypes were found for a morphological species, there was a large geographic distance between the sites for each haplotype. For example, of the two haplotypes of *N. hawaiiensis*, one was found on Keahole, the east slope of the Big Island in the Main Hawaiian Islands, while the other haplotype was found at Pioneer Bank, in the far NWHI (a distance of 1917 km). The same was true for *N. dichotoma. N. alata* and *N.* sp. 1, although not the closest related morphological species to each other, are the closest genetically to each other, and also span a large geographic range across the Archipelago (876 km).

As was seen with *N. alaskensis*, in all cases with two haplotypes, the two haplotypes of the given Hawaiian species also were separated by a significant depth interval (Figure 3). For example, the two haplotypes of *N. hawaiiensis* were separated by at least 1000 m depth, of *N. dichotoma* by about 500 m, and of *N. alata* vs. sp.1 by about 1100 m. The three Hawaiian haplotypes of the *N. macrocalyx/arbuscula/*sp. 2 clade were also spread across a depth gradient, with southeast Laysan having closely related haplotypes of the clade occurring at a gradient of depths on the same seamount.

**Figure 4:**
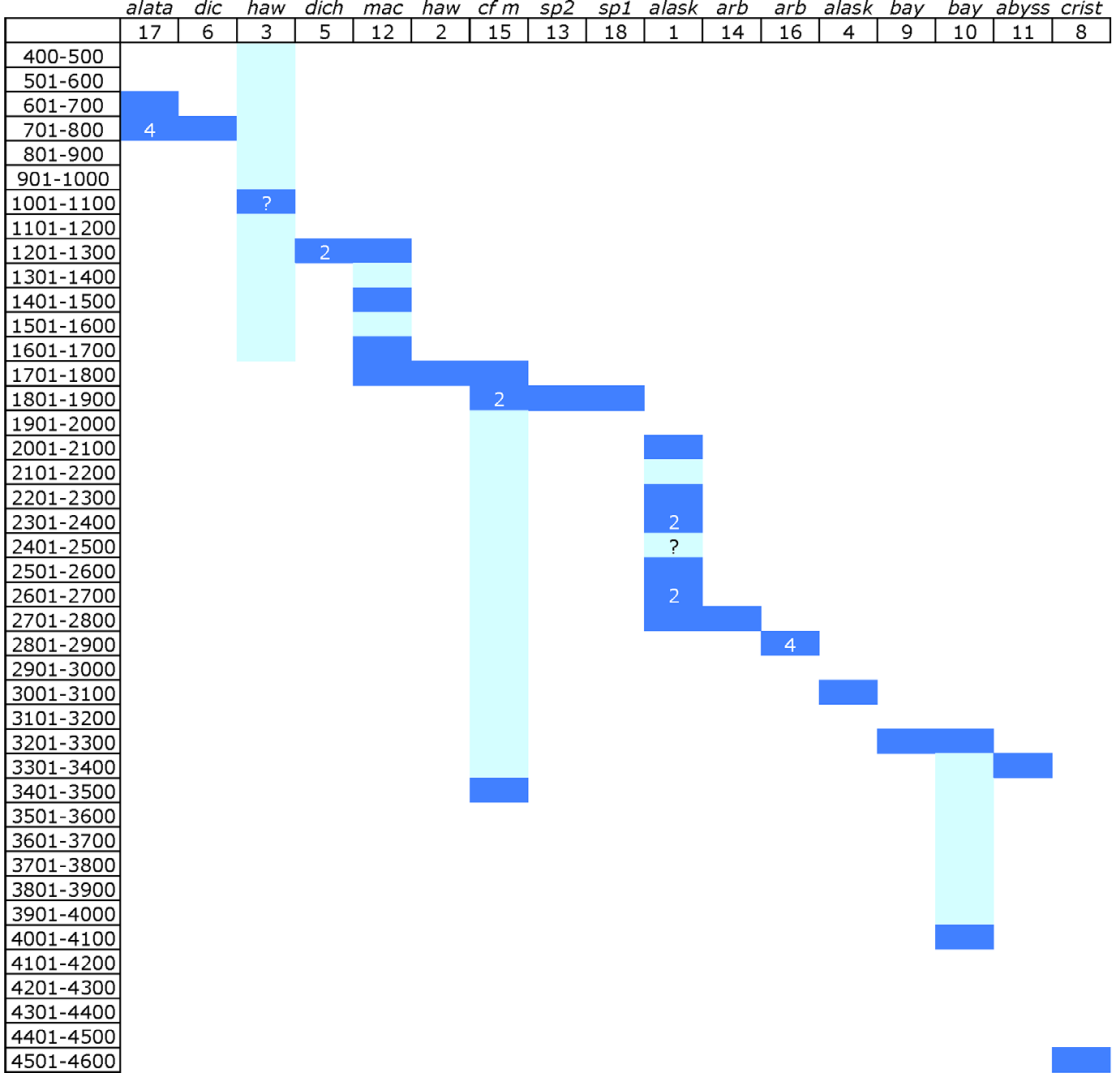
Depth distribution of *Narella* combination haplotypes sorted by minimum depth of occurrence. Combination haplotypes, as designated in Table 1, are given in the columns, along with the first three letters of the species name. 100 m depth bins are provided in the rows. Dark blue indicate an actual depth for a given haplotype, light blue indicates possible range for Haplotype 3 and is used to fill in the depth range for other columns. Numbers indicate number of individuals in a given depth range with that haplotype when the value is greater than 1. ? - indicates mean of possible range of depths for specimens for which depth was not recorded.

This depth difference of haplotypes within species led us to examine the depth distribution of all the *Narella* haplotypes further (Figure 4). This Figure is reminiscent of diagrams showing the distribution of species across depth e.g. [57] and suggests that depth may be structuring haplotype distributions.

### Phylogeny

**Figure 5:**
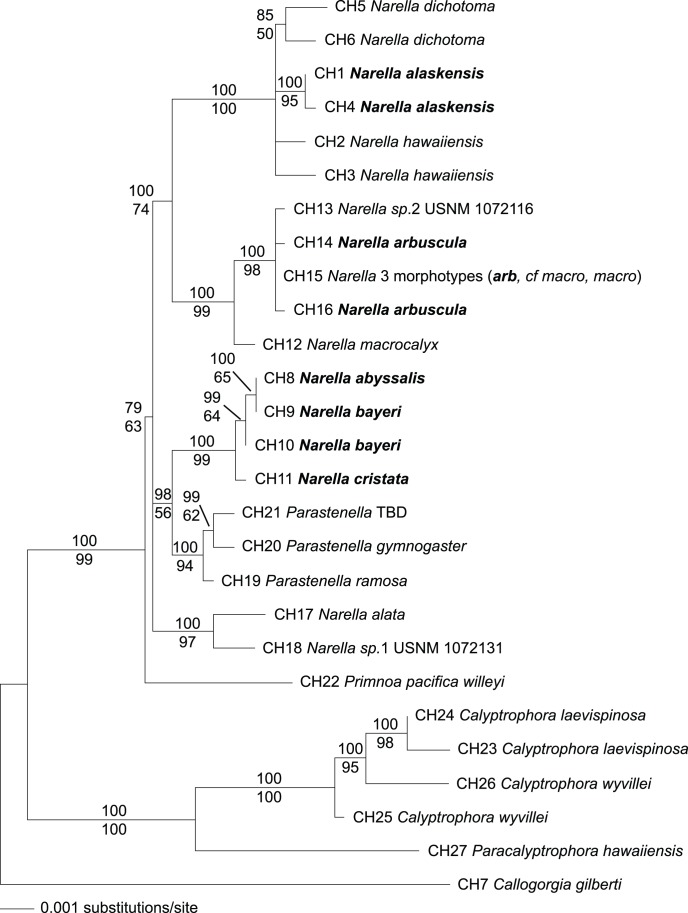
Maximum likelihood tree for taxa that amplified for all markers, based on 3348 nt alignment of combination haplotypes. Values above the line are Bayesian posterior probabilities and below the lines are maximum likelihood bootstrap values as percent of 1000 bootstraps. CH numbers correspond to combination haplotype designations as given in Table 1. *Narella* species names in bold indicate specimens collected in Alaska.

A phylogenetic tree constructed from the maximum likelihood heuristic search using combination sequences is shown in Figure 5. The alignment of the combination haplotypes was 3348 bp. The Bayesian consensus tree constructed using a 50% majority-rule consensus for 40,001 Bayesian tress minus the first 10,001 trees removed for burn-in had an identical branching order to the maximum likelihood tree (Fig. 5). There was no difference in topology between Bayesian runs 1 and 2 but the posterior probability value differed for the clade containing combination Haplotypes 5 and 6, (85 on run1 and 84 on run 2) as did the value for the clade containing all of the *Narella* and *Parastenella* (79 on run 1 and 78 on run 2). Bayesian and likelihood bootstrap values are shown.

Using the most divergent specimen, *Callogorgia,* as the root, the tree resolved a well-supported monophyletic clade including specimens from the genera *Narella*, *Parastenella* and *Primnoa*. A poorly supported clade within this (posterior probability 79, ML bootstrap 63) includes all specimens in the genus *Narella* as a paraphyletic group with species of *Parastenella*. Because of this ambiguity, we tested for monophyly of *Narella* using the Shimodaira-Hasegawa test [53]. With Narella constrained as monophyletic, the heuristic search yielded a single tree with *Parastenella* as a sister clade to *Narella* (not shown). This was compared to the topology shown in Figure 5. The SH test indicated that the unconstrained tree shown in Figure 5 was the best tree, but the p-value for the comparison to the constrained tree (0.289) was not significant. Although we cannot conclusively reject the monophyly of *Narella*, *Narella* and *Parastenella* are highly divergent morphologically. *Parastenella* is unique among the primnoid genera in having opercular scales offset from their marginal scales, marginal scales that are fluted in shape, and nematocyst pads on the inner face of the marginal scales. Furthermore, *Parastenella* differs from *Narella* in having a variable number of body wall scales arranged in 5–8 rows, whereas *Narella* has a fixed number of three or four pairs of body wall scales, and the polyps of *Parastenella* are oriented perpendicular to the branch, whereas in *Narella* they are facing downward [35]. The strong morphological divergence between *Narella* and *Parastenella*, along with the poor bootstrap and posterior probability support for the clade that include *Parastenella* and three *Narella* species, provides little support for paraphyly. Further genes and specimens will be needed to fully resolve this relationship.

Although the resolution of the phylogenetic tree is not sufficient to examine the deeper-branching between *Narella* clades to determine the evolutionary order of habitats, we can look at the geographic distribution and depth distribution within each of the well-resolved clades. Within each of the well-resolved clades of *Narella* are species and haplotypes from both Hawaii and Alaska (Figure 5), indicating that there was not a case of radiation within each seamount chain independently. The limited sampling for each species makes it hard to examine a depth pattern, but there also does not appear to be a strong tie of evolutionary history to depth (Figure 4 and 5). Markers which resolve the branching order within *Narella* will be required to fully resolve the evolutionary pattern with depth.

### Reassessment of Morphology Compared to the Molecular Results

There was one group of taxa for which the genetic data did not support the independent morphological data. The only group which confounded the genetic resolution of species were the four specimens which fell into combination Haplotype 15, which included 3 different morphotypes - two specimens of *Narella sp cf. macrocalyx* from the NWHI, one specimen of *Narella macrocalyx* from the NWHI (USNM 1072117), and one specimen of *Narella arbuscula* from Derickson (USNM 1080452). Cairns and Bayer [34], [54] noted in the description of *N. macrocalyx* that specimen USNM 1072117 was divergent from the other specimens. Re-examination of this specimens and the two *N. cf. macrocalyx* indicates that all three are the same morphotype. However, the *N. arbuscula* that falls into this clade, specimen USNM 1080452, was also re-examined and is indistinguishable morphologically from the other *N. arbuscula.* Thus, Haplotype 15 includes two morphotypes of *Narella* that will require additional markers to fully resolve.

*N. hawaiiensis* and *N. dichotoma* had the largest intraspecific distances between their respective haplotypes. Each of these specimens was re-examined to determine if the levels of genetic divergence between their respective haplotypes might be indicative of cryptic species or subspecies. No morphological characters could be found which would distinguish any of the specimens as subspecies. Since the genetic divergence between the haplotypes was less than the 0.5% cutoff suggested by McFadden et al [24], we do not have any genetic or morphological evidence at this time to support cryptic speciation.

## Discussion

The anthropogenic threats to seamounts make understanding levels of connectivity of seamount fauna an urgent priority. Deep-sea corals provide a good proxy for understanding the dynamics of connectivity on seamounts. They are also a critical group to understand for conservation and management in their own right as deep-sea corals have also been shown to be long-lived [58], [59], slow-growing [60], [61], recruitment limited [62], [63], and to act as ecosystem engineers [64], providing habitat for a suite of invertebrates and potentially for commercially important fishes [23], [65]–[67].

Understanding connectivity is key for conservation and a first step to determining connectivity is establishing the geographic range of species and the degree of species overlap between sites. Barcoding is a tool that may be useful in these efforts, provided there is some ground-truthing of what degree of variation indicates species. In assessing the possibility of using barcoding markers in basal metazoa, Huang *et al.* [68] recommend caution in relying on genetic markers as species proxies without “full taxonomic appraisal”. The work shown here is the beginning of efforts to develop a morphological taxonomic baseline that corresponds to the genetic variation for deep-sea octocorals, in a suite of genetic markers that have been used as potential barcoding proxies. Our results provide further insights into the utility of genetic markers as barcoding proxies for octocoral species as well as new insights into the distribution of seamount octocorals. They also provide a means of reevaluating the results of previous studies for which morphological data were not available.

### Evaluation of Markers

It is well established that mitochondrial markers in cnidarians are not as variable as in other taxa and that a given marker taken alone may not be sufficient to resolve morphological species (reviewed in[24], [68]–[70]). Our results are consistent with this finding and also indicate some markers that have been used as barcoding proxies or in octocoral phylogenetics, including NCR1 and ND2, do not have sufficient variation to distinguish between genera within the Primnoidae.

At the interspecific level within *Narella*, no single marker was able to resolve all of the morphological species. Although there were eight haplotypes for NCR1, only 1 of the morphological species of *Narella* was fully resolvable with this marker, all other *Narella* haplotypes were shared among more than one species or genus. Although MutS provided 11 haplotypes, these only provided full resolution for 6 of the 12 morphologic species, or about 50% of *Narella*. This is more than the 20% of *Alcyonium* morphological species that could be resolved with MutS in the McFadden *et al.* [24] study, but more comparable to the 42% of *Alcyonium* species which could be resolved when only Atlantic specimens were considered.

There was a much better success rates when all of the markers were taken together, however even with this ∼3350 of the mitochondrial genome, including what are thought to be the most variable regions, only 10 of 12 of the morphological species of *Narella* or about 83%, could be fully resolved, i.e. having haplotypes that were unique only to a given species. This shows greater resolution than McFadden *et al.* [24], who were able to resolve only 4 of 10 morphological species of *Alcyonium* using the combined MutS and COI+ “extended barcode”.

Although the combination haplotypes had the greatest resolving power, not all of the markers contributed to this resolving power. Removing NCR1 and/or ND6 from the sequences provided the same number of fully resolved species. Although ND2 had a smaller range of variation and genetic divergence than ND6, removing ND2 and using just the COI+ and MutS markers, the equivalent of the “extended barcode” suggested by McFadden *et al.* [24], reduced the number of fully resolved species by one.

Clearly a nuclear marker will be needed to attain 100% resolution of species. Until this marker is discovered, we recommend using a combination of the MutS and COI+ markers (the “extended” barcode of [24]) along with the ND2 marker. Taken together these can resolve about 83% of species using a character-based approach. This is an increase over the ∼40–60% based on MutS and COI+ alone ([24]and Table 3, respectively) and also better than the 50% resolution currently possible based on morphological taxonomy (discussed in Introduction).

### Implications for Previous Studies and for Seamount Connectivity

Although we could not resolve all morphological species, our results provide further insights into the variation in markers that have been used in deep-sea octocorals as proxies for species. For example, these are among the first results to indicate that a given octocoral species may have more than 1 haplotype for ND6, COI+, or MutS. Given this result, interpreting each MutS haplotype as a species (e.g. [10]) may inflate the species estimate, although this is somewhat countered by a portion of haplotypes for each marker being shared between species, which would underestimate the total diversity. This confusion of haplotypes may also impact the interpretation of species ranges.

Previous genetic work on deep-sea octocorals has focused on seamount specimens and has shown that octocoral haplotypes that occur on seamounts are very widespread [7], [10]. The assumption that these haplotypes correspond to morphological species, therefore implying that species are also widespread, has contributed to shifting the paradigm of seamount isolation, even though the authors of the genetic work clearly caution that the level of genetic variation in the markers used for their studies have not been tied to a particular taxonomic level. Our results provide some of the first baseline data to more clearly determine what the levels of genetic variation observed in those two studies actually correspond to taxonomically. Assuming the levels of genetic variation seen in *Narella* and closely related primnoid genera are comparable to what is found in other deep-sea octocoral taxa, the results shown here suggest that the markers used in these previous studies were not accurate indicators of species-level variation.

Smith *et al.* [7] focused their efforts on two markers, the more variable of which, NCR1, we find here to not have sufficient resolution to distinguish between species in a genus, or even *between genera* within Primnoidae. For example, the examination of the distribution of NCR1 haplotypes within *Narella* (Figure 1) indicates at least 1 haplotype that occurs at all the sites studied, and several other NCR1 haplotypes also have very broad distributions. Relying solely on this marker, we would conclude a broad distribution for *Narella* species. In contrast, in Figures 2, 3 and 4, which include the combined haplotypes, and still only resolves 83% of species, we see a very different pattern, inferring a much narrower distribution for *Narella* species. Assuming levels of variation in these markers are comparable for Isididae, our results suggest that Smith *et al.* [7] were looking at genetic variation above the species level. Following this line of reasoning, what their results likely show is that *genera* within Isididae, are geographically widespread, rather than species. As most genera and families in any deep-sea taxon are widespread, genera and subfamilies of octocorals being widespread is neither surprising nor incongruent with the potential for seamount endemicity at the species or population level.

Thoma *et al.* [10] similarly used MutS as a proxy for species, in a wider range of deep-sea octocoral families. In their work, they made the stated assumption that each haplotype corresponded to a morphological species. However our results indicate that not only can well-defined morphological species have more than one MutS haplotype but also that well-defined species or morphotypes may share haplotypes. As already pointed out, this may affect diversity estimates, but more significantly, it can impact estimates of species ranges. Our results indicate that although 11 haplotypes were found in MutS for 12 morphotypes of *Narella*, in fact 2 of the 11 haplotypes were shared between multiple species or morphotypes. Therefore, the lack of endemism of haplotypes to a seamount found by Thoma *et al.* [10] could just as easily be attributed to a lack of resolving power of their chosen marker. Based on this, Thoma *et al.* [10] were likely underestimating species richness and overestimating species distributions. Thus again we see a result for octocoral distributions that is neither surprising nor incongruent with the potential for endemicity at the species or population level.

In contrast to these studies, with higher resolution markers we find that the haplotypes for *Narella* have relatively narrow depth and geographic ranges (Figures 2, 3, 4), albeit sampled over a only a single region. Geographic distance between locations appears to play some role in species differentiation since for all morphologically well-resolved species with two haplotypes, the two haplotypes had non-overlapping geographic ranges. However, there is a more pronounced signal of depth differentiation between the two haplotypes of a given species (Figure 3). There was also a gradation of haplotypes with depth within *Narella* (Figure 4). Genetic structure with depth has been shown in other deep-sea invertebrate taxa [71]–[75] and also among deep-sea octocoral populations [76]. Geographic and depth isolation of haplotypes may also be an indication of subspecies and cryptic speciation. However, as discussed above, we did not find sufficient genetic divergence to suggest cryptic speciation and there were no morphological differences between specimens that had more than one haplotype for a given species.

### Implications for Seamount Conservation and Management

With the increased resolving power of genetic markers, we are seeing a more restricted distribution for deep-sea octocorals on seamounts, implying that markers that resolve 100% of species may indicate an even more restricted distribution. Unfortunately, we are still not to that 100% point, and are just scratching the surface of intraspecific variation using these markers. Because of this, we would argue that the degree of species overlap for seamount octocorals has not yet been resolved, and therefore, the hypothesis of seamount connectivity has not been addressed in existing studies. Additionally, using existing mitochondrial markers, we do not have the power to even test the hypothesis, let alone refute it. Nuclear markers such as microsatellites or SNPs, or next-generation sequencing approaches will likely provide a more accurate determination of the degree of genetic isolation of seamount populations. Given the lack of current tests of genetic isolation and the tendency towards narrower ranges with increased resolution, a precautionary approach is warranted when considering the degree of genetic isolation of a given seamount or seamount chain in conservation management.

Additionally, the finding of genetic differentiation with depth supports a growing body of evidence that seamount populations may have genetic structure with depth on a single seamount or oceanic island feature [32], [75], [76]. This is further supported by findings of changes in community structure with depth on seamounts [14], [77]. Given this, we suggest that treating seamounts as homogenous features may be an incorrect assumption. We recommend that the potential depth zonation of a seamount also be considered in scientific sampling in order to compare features and also to assess conservation and management issues.

## Supporting Information

Table S1 **GenBank Accession numbers for each species for each marker.**
(DOCX)

Table S2 **Variable base positions within** ***Narella*** **sequences.** CH indicates combination haplotypes as given in Table 1. Base positions are positions in the overall alignment, with marker they fall into indicated in the top row. – indicates an alignable gap position.
(DOCX)
