# Supplementary material for: Comparing Molecular Variation to Morphological Species Designations in the Deep-Sea Coral Narella Reveals New Insights into Seamount Coral Ranges
Source: PLoS One. 2012 Sep 27;7(9):e45555. doi: 10.1371/journal.pone.0045555 (PMC3459954; doi:10.1371/journal.pone.0045555)
Supplement: Table S1 — GenBank Accession numbers for each species for each marker. (DOCX) [file pone.0045555.s001.docx]

Table S1.

| **USNM**  **Numbers** | **Published ID** | **NCR1** | **ND6** | **ND2** | **COI+** | **MutS** |
| --- | --- | --- | --- | --- | --- | --- |
| 1075468, 1075471, 1080453, 1075469 | *Narella alaskensis* Cairns & Baco, 2007 | JX566859 | JX566834 | JX561198 | JX561153 | JX561172 |
| 1080454 | *Narella alaskensis* Cairns & Baco, 2007 | JX566859 | JX566834 | JX561197 | JX561153 | JX561172 |
| 1072109 | *Narella hawaiiensis* Cairns & Bayer, 2008 | JX566864 | JX566850 | JX561199 | JX561154 | JX561180 |
| 1071215 | *Narella hawaiiensis* Cairns & Bayer, 2008 | JX566860 | JX566850 | JX561208 | JX561148 | JX561184 |
| 1072111, 1072112 | *Narella dichotoma Cairns & Bayer, 2008* | JX566858 | JX566842 | JX561207 | JX561155 | JX561182 |
| 1071422 | *Narella dichotoma Cairns & Bayer, 2008* | JX566858 | JX566848 | JX561207 | JX561155 | JX561191 |
| 1071418, 1071421, 1071420, 1071419 | *Narella alata Cairns & Bayer, 2008* | JX566851 | JX566829 | JX561200 | JX561147 | JX561168 |
| 1072131 | *Narella sp. 1 (morph closest to dichotoma)* | JX566852 | JX566830 | JX561206 | JX561146 | JX561179 |
| 1080450 | *Narella abyssalis Cairns & Baco, 2007* | JX566865 | JX566831 | JX561192 | JX561167 | JX561169 |
| 1080447 | *Narella bayeri Cairns & Baco, 2007* | JX566866 | JX566832 | JX561193 | JX561145 | JX561170 |
| 1080448, 1080446 | *Narella bayeri Cairns & Baco, 2007* | JX566867 | JX566832 | JX561193 | JX561145 | JX561170 |
| 1080449 | *Narella cristata Cairns & Baco, 2007* | JX566868 | JX566833 | JX561194 | JX561166 | JX561171 |
| 1072118, 1072133, 1072122, 1072105 | *Narella macrocalyx Cairns & Bayer, 2008* | JX566857 | JX566835 | JX561202 | JX561163 | JX561181 |
| 1072116 | *Narella sp. 2* | JX566861 | JX566836 | JX561201 | JX561149 | JX561173 |
| 1072108, 1072103, 1072117 | *Narella sp. cf. macrocalyx* | JX566862 | JX566837 | JX561203 | JX561150 | JX561174 |
| 1080452 | *Narella arbuscula Cairns & Baco, 2007* | JX566863 | JX566838 | JX561204 | JX561151 | JX561175 |
| 1080451 | *Narella arbuscula Cairns & Baco, 2007* | JX566863 | JX566838 | JX561204 | JX561158 | JX561175 |
| 1075465, 1075466, 1075467 | *Narella arbuscula Cairns & Baco, 2007* | JX566863 | JX566838 | JX561204 | JX561151 | JX561176 |
| 1154063 | *Callogorgia gilberti* | JX566856 | JX566841 | JX561212 | JX561164 | JX561190 |
| 1075379 | *Parastenella ramosa* | JX566853 | JX566845 | JX561209 | JX561156 | JX561185 |
| 1082620 or 1082624 | *Parastenella gymnogaster* | JX566854 | JX566846 | JX561195 | JX561157 | JX561188 |
| 1082639 | *Parastenella ramosa* | JX566853 | JX566849 | JX561196 | JX561156 | JX561187 |
| 1075478 | *Primnoa pacifica willeyi Hickson, 1915* | JX566855 | JX566840 | JX561205 | JX561160 | JX561178 |
| 1082615, 1082617 | *Calyptrophora laevispinosa* | JX566872 | JX566839 | JX561213 | JX561162 | JX561177 |
| 1075472, 1082616 | *Calyptrophora laevispinosa* | JX566872 | JX566839 | JX561213 | JX561165 | JX561177 |
| 1071947, 1071423 | *Calyptrophora wyvillei Wright 1885* | JX566871 | JX566847 | JX561214 | JX561152 | JX561186 |
| 1072130 | *Calyptrophora wyvillei Wright 1885* | JX566869 | JX566843 | JX561211 | JX561159 | JX561183 |
| 1072135, 1071245 | *Paracalyptrophora hawaiiensis Cairns 2009* | JX566870 | JX566844 | JX561210 | JX561161 | JX561189 |
