# Supplementary material for: Comparing Molecular Variation to Morphological Species Designations in the Deep-Sea Coral Narella Reveals New Insights into Seamount Coral Ranges
Source: PLoS One. 2012 Sep 27;7(9):e45555. doi: 10.1371/journal.pone.0045555 (PMC3459954; doi:10.1371/journal.pone.0045555)
Supplement: Table S2 — Variable base positions within Narella sequences. CH indicates combination haplotypes as given in Table 1. Base positions are positions in the overall alignment, with marker they fall into indicated in the top row. – indicates an alignable gap position. (DOCX) [file pone.0045555.s002.docx]

Table S2.

|  | **NCR1** | | | | | | | | | | **ND6** | | | | | | |
| --- | --- | --- | --- | --- | --- | --- | --- | --- | --- | --- | --- | --- | --- | --- | --- | --- | --- |
|  | 87 | 92 | 93 | 94 | 95 | 96 | 111 | 210 | 214 | 279 | 295 | 444 | 445 | 502 | 529 | 691 | 790 |
| CH5 *N. dichotoma* | A | T | T | A | C | C | A | C | A | C | A | T | C | C | A | C | A |
| CH6 *N. dichotoma* | A | T | T | A | C | C | A | C | A | C | T | T | A | C | C | C | A |
| CH1 *N. alaskensis* | A | T | T | A | C | C | A | C | A | A | T | T | A | C | A | C | A |
| CH4 N. alaskensis | A | T | T | A | C | C | A | C | A | A | T | T | A | C | A | C | A |
| CH2 N. *hawaiiensis* | C | T | T | A | C | C | A | C | A | A | T | T | A | C | A | C | A |
| CH3 N. *hawaiiensis* | A | T | T | A | C | C | A | C | A | A | T | T | A | C | A | C | A |
| CH13 *N. sp.* 2 | C | T | T | A | C | C | A | T | C | A | T | T | A | C | A | C | A |
| CH14 *N. arbuscula* | C | T | T | A | C | C | A | T | C | A | T | T | A | C | A | C | A |
| CH15 *N.* 3 morphs | C | T | T | A | C | C | A | T | C | A | T | T | A | C | A | C | A |
| CH16 *N. arbuscula* | C | T | T | A | C | C | A | T | C | A | T | T | A | C | A | C | A |
| CH12 *N. macrocalyx* | C | C | T | A | C | C | A | T | C | A | T | T | A | C | A | C | A |
| CH8 *N. abyssalis* | A | - | - | - | - | - | C | T | A | A | T | T | A | C | A | T | C |
| CH9 *N. bayeri* | A | - | - | - | - | - | C | T | A | A | T | T | A | C | A | T | C |
| CH10 *N. bayeri* | A | - | - | - | - | - | A | T | A | A | T | T | A | C | A | T | C |
| CH11 *N. cristata* | A | - | - | - | - | - | A | T | A | A | T | T | A | C | A | T | C |
| CH17 *N. alata* | A | T | T | A | C | C | A | T | A | A | T | T | A | A | A | T | C |
| CH18 *N. sp*. 1 | A | T | T | A | C | C | A | T | A | A | T | T | A | A | A | T | C |

|  |  |  |  |  |  |  |  |  |  |  |  |  |  |  |  |  |  |  |  |  |  |  |  |
| --- | --- | --- | --- | --- | --- | --- | --- | --- | --- | --- | --- | --- | --- | --- | --- | --- | --- | --- | --- | --- | --- | --- | --- |
|  | **ND2** | | | | | | | | | | | | | | | | | | | | | | |
|  | 890 | 917 | 964 | 1094 | 1113 | 1239 | 1263 | 1331 | 1332 | 1333 | 1334 | 1335 | 1336 | 1337 | 1338 | 1339 | 1340 | 1341 | 1342 | 1404 | 1416 | 1469 | 1528 |
| CH5 *N. dichotoma* | T | T | A | C | T | C | C | C | A | C | C | T | A | A | T | A | A | C | C | C | T | A | A |
| CH6 *N. dichotoma* | T | T | A | C | T | C | C | C | A | C | C | T | A | A | T | A | A | C | C | C | T | A | A |
| CH1 *N. alaskensis* | T | T | A | C | T | C | C | - | - | - | - | - | - | - | - | - | - | - | - | C | T | A | A |
| CH4 N. alaskensis | T | T | A | C | T | C | C | - | - | - | - | - | - | - | - | - | - | - | - | A | T | A | A |
| CH2 N. *hawaiiensis* | T | T | A | C | T | C | C | - | - | - | - | - | - | - | - | - | - | - | - | C | T | A | A |
| CH3 N. *hawaiiensis* | T | - | A | C | T | C | C | C | A | C | C | T | A | A | T | A | A | C | C | C | C | A | A |
| CH13 *N. sp.* 2 | T | T | C | A | C | C | T | C | A | C | C | T | A | A | T | A | A | C | C | C | T | A | A |
| CH14 *N. arbuscula* | T | T | A | A | C | C | T | C | A | C | C | T | A | A | T | A | A | C | C | C | T | A | A |
| CH15 *N.* 3 morphs | T | T | A | A | C | C | T | C | A | C | C | T | A | A | T | A | A | C | C | C | T | A | A |
| CH16 *N. arbuscula* | T | T | A | A | C | C | T | C | A | C | C | T | A | A | T | A | A | C | C | C | T | A | A |
| CH12 *N. macrocalyx* | T | T | A | A | C | C | T | C | A | C | C | T | A | A | T | A | A | C | C | C | T | A | A |
| CH8 *N. abyssalis* | T | T | A | A | T | C | T | - | - | - | - | - | - | - | - | - | - | - | - | C | T | A | A |
| CH9 *N. bayeri* | T | T | A | A | T | C | T | - | - | - | - | - | - | - | - | - | - | - | - | C | T | A | A |
| CH10 *N. bayeri* | T | T | A | A | T | C | T | - | - | - | - | - | - | - | - | - | - | - | - | C | T | A | A |
| CH11 *N. cristata* | T | T | A | A | T | C | T | - | - | - | - | - | - | - | - | - | - | - | - | C | T | A | A |
| CH17 *N. alata* | - | T | A | A | T | T | T | C | A | C | C | T | A | A | T | A | A | C | C | C | T | C | C |
| CH18 *N. sp*. 1 | T | T | A | A | T | T | T | C | A | C | C | T | A | A | T | A | A | C | C | C | T | A | A |
|  |  |  |  |  |  |  |  |  |  |  |  |  |  |  |  |  |  |  |  |  |  |  |  |

|  |  |  |  |  |  |  |  |  |  |  |  |  |  |  |  |  |  |  |  |  |  |  |  |  |  |  |  |  |  |  |  |
| --- | --- | --- | --- | --- | --- | --- | --- | --- | --- | --- | --- | --- | --- | --- | --- | --- | --- | --- | --- | --- | --- | --- | --- | --- | --- | --- | --- | --- | --- | --- | --- |
|  | **COI+** | | | | | | | | | | | | | | | | | | | | | | | | | | | | | | |
|  | 1692 | 1718 | 1748 | 1749 | 1796 | 1980 | 1982 | 1988 | 2040 | 2061 | 2136 | 2249 | 2265 | 2372 | 2374 | 2375 | 2376 | 2377 | 2378 | 2379 | 2380 | 2381 | 2382 | 2383 | 2384 | 2385 | 2386 | 2391 | 2412 | 2423 | 2462 |
| CH5 *N. dichotoma* | A | A | C | A | T | T | T | A | A | T | C | C | T | C | A | T | C | C | A | A | C | C | T | A | C | A | T | A | T | T | T |
| CH6 *N. dichotoma* | A | A | C | A | T | T | T | A | A | T | C | C | T | C | A | T | C | C | A | A | C | C | T | A | C | A | T | A | T | T | T |
| CH1 *N. alaskensis* | A | A | C | A | T | T | T | A | A | T | C | C | T | C | A | T | C | C | A | A | C | C | T | A | C | A | T | A | T | T | T |
| CH4 N. alaskensis | A | A | C | A | T | T | T | A | A | T | C | C | T | C | A | T | C | C | A | A | C | C | T | A | C | A | T | A | T | T | T |
| CH2 N. *hawaiiensis* | A | A | C | A | T | T | T | A | A | T | C | C | T | C | A | T | C | C | A | A | C | C | T | A | C | A | T | A | T | T | T |
| CH3 N. *hawaiiensis* | A | A | C | A | C | T | T | A | A | T | C | C | T | C | A | T | C | C | A | A | C | C | T | A | C | A | T | A | T | T | T |
| CH13 *N. sp.* 2 | A | A | C | A | T | T | C | A | A | C | T | C | C | C | A | T | C | C | A | A | C | C | T | A | C | A | T | A | T | C | T |
| CH14 *N. arbuscula* | A | A | C | A | T | T | C | A | A | C | T | C | C | C | A | T | C | C | A | A | C | C | T | A | C | A | C | A | T | C | T |
| CH15 *N.* 3 morphs | A | A | C | A | T | T | C | A | A | C | T | C | C | C | A | T | C | C | A | A | C | C | T | A | C | A | T | A | T | C | T |
| CH16 *N. arbuscula* | A | A | C | A | T | T | C | A | A | C | T | C | C | C | A | T | C | C | A | A | C | C | T | A | C | A | T | A | T | C | T |
| CH12 *N. macrocalyx* | A | A | C | A | T | T | T | A | A | C | T | C | T | C | A | T | C | C | A | A | C | C | T | A | C | A | T | A | T | C | T |
| CH8 *N. abyssalis* | A | C | T | A | T | T | T | C | A | T | T | C | T | - | - | - | - | - | - | - | - | - | - | - | - | - | T | A | C | T | Y |
| CH9 *N. bayeri* | A | C | T | A | T | T | T | C | A | T | T | C | T | C | A | T | C | C | A | A | C | C | T | A | C | C | T | A | C | T | T |
| CH10 *N. bayeri* | A | C | T | A | T | T | T | C | A | T | T | C | T | C | A | T | C | C | A | A | C | C | T | A | C | C | T | A | C | T | T |
| CH11 *N. cristata* | A | C | T | C | T | T | T | C | A | T | T | C | T | C | A | T | C | C | A | A | C | C | T | A | C | C | T | A | T | T | T |
| CH17 *N. alata* | C | A | C | A | T | C | T | A | C | T | T | A | T | C | A | T | C | C | A | A | C | C | T | A | C | A | T | A | T | T | T |
| CH18 *N. sp*. 1 | C | C | C | A | T | C | T | A | A | T | T | A | T | C | A | T | C | C | A | A | C | C | T | A | C | A | T | C | T | T | T |

|  |  |  |  |  |  |  |  |  |  |  |  |  |  |  |  |  |  |  |  |  |  |  |  |  |  |  |
| --- | --- | --- | --- | --- | --- | --- | --- | --- | --- | --- | --- | --- | --- | --- | --- | --- | --- | --- | --- | --- | --- | --- | --- | --- | --- | --- |
|  | **MutS** | | | | | | | | | | | | | | | | | | | | | | | | | |
|  | 2504 | 2568 | 2576 | 2614 | 2616 | 2626 | 2663 | 2731 | 2734 | 2747 | 2752 | 2762 | 2827 | 2880 | 2885 | 2943 | 2949 | 3039 | 3127 | 3162 | 3223 | 3240 | 3263 | 3318 | 3322 | 3336 |
| CH5 *N. dichotoma* | T | A | A | C | C | A | A | C | A | A | C | A | C | C | A | C | C | A | C | A | C | A | C | A | A | A |
| CH6 *N. dichotoma* | T | A | C | C | C | A | A | T | A | A | C | A | C | C | A | C | C | A | C | A | C | A | C | A | A | - |
| CH1 *N. alaskensis* | T | A | A | C | C | A | A | T | A | A | C | A | C | A | A | A | T | A | C | A | C | C | C | A | A | A |
| CH4 N. alaskensis | T | A | A | C | C | A | A | T | A | A | C | A | C | A | A | A | T | A | C | A | C | C | C | A | A | A |
| CH2 N. *hawaiiensis* | T | A | A | C | C | C | A | T | A | A | C | C | C | C | A | A | C | A | C | A | C | A | C | A | A | A |
| CH3 N. *hawaiiensis* | T | A | A | C | C | A | A | T | A | C | C | A | C | C | A | A | C | A | T | A | C | A | C | A | A | A |
| CH13 *N. sp.* 2 | C | A | A | C | A | A | C | T | A | A | T | A | C | C | C | C | C | A | C | A | T | A | C | A | A | A |
| CH14 *N. arbuscula* | C | A | A | C | A | A | C | T | A | A | T | A | C | C | C | C | C | A | C | A | T | A | C | A | A | A |
| CH15 *N.* 3 morphs | C | A | A | C | A | A | C | T | A | A | T | A | C | C | C | C | C | A | C | A | T | A | C | A | A | A |
| CH16 *N. arbuscula* | C | A | A | C | A | A | C | T | A | A | T | A | C | C | C | C | C | C | C | A | T | A | C | A | A | A |
| CH12 *N. macrocalyx* | T | A | A | C | A | A | C | T | A | A | T | A | C | C | A | C | C | A | C | C | T | A | C | A | A | A |
| CH8 *N. abyssalis* | T | T | A | C | A | A | C | T | A | A | T | A | A | C | A | C | C | A | C | A | T | A | C | C | A | A |
| CH9 *N. bayeri* | T | T | A | C | A | A | C | T | A | A | T | A | A | C | A | C | C | A | C | A | T | A | C | C | A | A |
| CH10 *N. bayeri* | T | T | A | C | A | A | C | T | A | A | T | A | A | C | A | C | C | A | C | A | T | A | C | C | A | A |
| CH11 *N. cristata* | T | T | A | C | A | A | C | T | A | A | T | A | A | C | A | C | C | A | C | A | T | A | C | C | A | A |
| CH17 *N. alata* | T | A | A | A | A | A | C | T | C | A | T | A | A | C | A | C | C | A | C | A | T | A | A | A | A | A |
| CH18 *N. sp*. 1 | T | A | A | C | A | A | C | T | C | A | T | A | A | C | A | C | C | A | C | A | T | C | C | A | C | A |
